# A novel multi-drug metronomic chemotherapy significantly delays tumor growth in mice

**DOI:** 10.1186/s12967-016-0812-1

**Published:** 2016-02-24

**Authors:** Maria Tagliamonte, Annacarmen Petrizzo, Maria Napolitano, Antonio Luciano, Domenica Rea, Antonio Barbieri, Claudio Arra, Piera Maiolino, Marialina Tornesello, Gennaro Ciliberto, Franco M. Buonaguro, Luigi Buonaguro

**Affiliations:** Laboratory of Molecular Biology and Viral Oncology, Istituto Nazionale per lo Studio e la Cura dei Tumori, “Fondazione Pascale” - IRCCS, Naples, Italy; Laboratory of Clinical Immunology, Istituto Nazionale per lo Studio e la Cura dei Tumori, “Fondazione Pascale” - IRCCS, Naples, Italy; Animal Facility, Istituto Nazionale per lo Studio e la Cura dei Tumori, “Fondazione Pascale” - IRCCS, Naples, Italy; Pharmacy Unit, Istituto Nazionale per lo Studio e la Cura dei Tumori, “Fondazione Pascale” - IRCCS, Naples, Italy; Scientific Direction, Istituto Nazionale per lo Studio e la Cura dei Tumori, “Fondazione Pascale” - IRCCS, Naples, Italy

**Keywords:** Immunotherapy, Daily metronomic chemotherapy, Treg

## Abstract

**Background:**

The tumor immunosuppressive microenvironment represents a major obstacle to an effective tumor-specific cellular immune response.

**Methods:**

In the present study, the counterbalance effect of a novel metronomic chemotherapy protocol on such an immunosuppressive microenvironment was evaluated in a mouse model upon sub-cutaneous ectopic implantation of B16 melanoma cells. The chemotherapy consisted of a novel multi-drug cocktail including taxanes and alkylating agents, administered in a daily metronomic fashion. The newly designed strategy was shown to be safe, well tolerated and significantly efficacious.

**Results:**

Treated animals showed a remarkable delay in tumor growth and prolonged survival as compared to control group. Such an effect was directly correlated with CD4^+^ T cell reduction and CD8^+^ T cell increase. Furthermore, a significant reduction in the percentage of both CD25^+^FoxP3^+^ and CD25^+^CD127^low^ regulatory T cell population was found both in the spleens and in the tumor lesions. Finally, the metronomic chemotherapy induced an intrinsic CD8^+^ T cell response specific to B16 naturally expressed Trp2 TAA.

**Conclusion:**

The novel multi-drug daily metronomic chemotherapy evaluated in the present study was very effective in counterbalancing the immunosuppressive tumor microenvironment. Consequently, the intrinsic anti-tumor T cell immunity could exert its function, targeting specific TAA and significantly containing tumor growth. Overall, the results show that this represents a promising adjuvant approach to significantly enhance efficacy of intrinsic or vaccine-elicited tumor-specific cellular immunity.

## Background

Containment of tumor growth by cellular immunity is significantly hampered by intrinsic immunosuppressive mechanisms. CD4^+^CD25^+^FoxP3^+^ regulatory T cells (Tregs) and myeloid-derived suppressor cells (MDSCs) are the main cell types contrasting the effective anti-tumor cellular immunity.

Tregs represent about 4 % of peripheral CD4^+^T cells in both humans and mice, playing a relevant physiological control mechanism on the effector immune response. Indeed, they promote peripheral immune tolerance by suppressing self-antigen reactive T cells preventing the emergence of phathological autoimmune response [1]. On the contrary, in cancer patients, high percentage of Tregs in peripheral blood as well as in tumor site is associated with tumor progression and poor prognosis [2, 3]. Numerous studies have demonstrated that Tregs cause a reduction of antitumor immunity by suppressing NK and T cell responses through various mechanisms, including the expression of the inhibitory checkpoint regulator cytotoxic T lymphocyte-associated protein 4 (CTLA4) on their surface and the production of immunosuppressive mediators such as IL-10 and transforming growth factor β1 (TGFβ1) [4–6].

Myeloid-derived suppressor cells (MDSCs) are a heterogeneous population of myeloid cells, which suppress innate and adaptive antitumor immune response by favoring the recruitment and expansion of Tregs, as well as by producing high levels of L-arginase, reactive oxygen species (ROS), inducible nitrogen oxide synthase (iNOS), and various immunosuppressive cytokines. Overall, these enzymes and soluble mediators promote neo angiogenesis and allow the escape of immune cells from immune surveillance promoting disease progression [7–10]. Moreover, the accumulation of such MDSCs in the peripheral blood and within neoplastic lesions has been associated with poor prognosis in a large number of cancer patients [11].

Such an intra-tumoral immunosuppressive environment is one of the major foundation of the tumor escaping the immune mediated control as well as the unsatisfactory results observed in cancer immunotherapy clinical trials [12–15].

Treatments aiming at limiting these immunosuppressive cell populations should be taken into consideration in order to restore antitumor immune responses and improve cancer vaccine efficacy [16].

Low-dose or metronomic chemotherapeutic regimens have been shown to selectively kill immunosuppressive cell populations. This would eventually result in dramatic improvement in both the intrinsic immune containment of tumor growth and the immune response elicited by immunotherapy strategies.

Conventional chemotherapeutic agents were developed for their capacity to directly kill malignant cells at a maximal tolerated dose (MTD), the highest amount of the drug associated with tolerable toxicity. More recently, alternative schedules of treatment have been proposed and evaluated for such agents. Repetitive administration of chemotherapeutics at low dosage (so-called, metronomic chemotherapy) has been shown to induce an antiangiogenic activity associated with strong anti-neoplastic effects in absence of toxicity (reviewed in [17]). Additionally, low dose of metronomic chemotherapy have been shown to promote the expression of MHC class I molecules on the surface of cancer cells, facilitating their antigen-dependent killing mediated by CD8^+^ cytotoxic T cells (CTL) [18]. Moreover, metronomic chemotherapy induce in cancer cells an “immunogenic death”resulting in release of immunostimulatory factors (reviewed in [19]).

Chemotherapeutics can convert the tumor microenvironment into a site permissive for vaccination by enhancement of antigen density and accumulation of dendritic cells [20]. Moreover, pretreatment with metronomic chemotherapy can enhance the immunization efficacy by promoting the maturation and activation of dendritic cells (DCs) in vitro, in turn stimulating the proliferation of CD4 and CD8 T cells along with an increased production of interferon γ (IFN-γ) [18].

In this framework, it has been shown that metronomic chemotherapy with cyclophosphamide (CTX) or paclitaxel (PTX) in single or daily administration may significantly reduce the number of regulatory T cells [3, 21]. Such an effect results in improved T cell–mediated antitumor responses in both preclinical and clinical studies [22, 23] as well as improved immunogenicity of vaccines [24–26]. A combination of CTX, PTX and doxorubicin has been previously shown to enhance the immune response to vaccine when administered in a single administration schedule (i.e. 1 day before or 7 days after vaccination) [27]. Docetaxel (DTX) has been previously shown to increase antigen-specific T cell responses when combined to a vaccine with a schedule different from daily [28].

In this perspective, the overall objective of this study was to evaluate the immunomodulatory effects of a novel multi drugs metronomic chemotherapy in an aggressive therapeutic setting based on sub-cutaneous ectopic implantation of B16 melanoma cells. In particular, the metronomic chemotherapy included a multi-drug cocktail including taxanes (DTX and PTX) and alkylating (CTX) agents, aiming at hitting different targets of the immune suppressive tumor microenvironment [29].

The newly designed strategy in the present study was shown to be safe, well tolerated and significantly efficacious in both delaying tumor growth and prolonging animal survival. Such anti-tumor biological effects directly correlated with reduction of the immune suppressive Tregs cell population.

These results are highly promising for an effective counterbalancing of the intra-tumor suppressive micro-environment and pave way to new adjuvant strategies to significantly improve immunotherapeutic treatments.

## Methods

### Cell line and mice

C57BL/6 (H-2b MHC) female mice, 8 week old, were purchased from Harlan (Udine, Italy). All animals were housed at the Animal Facility of the Istituto Nazionale Tumori “Pascale” (Naples, Italy). Mice were maintained under specific pathogen-free conditions, and all procedures were in accordance with recommendations for the proper use and care of laboratory animals.

Mouse melanoma B16F10 (ATCC, CRL-6323) cells were cultured in DMEM supplemented with 10 % heat inactivated FBS, 100 U/ml penicillin and 100 mg/ml streptomycin (Invitrogen, Carlsbad, CA) at 37 °C with 5 % CO_2_. Cells were tested for mycoplasma before inoculation in mice.

### Antibodies for flow cytometry

Rabbit polyclonal antibody against calreticulin (PA3-900) was purchased from ThermoFisher (Rockford, IL, USA). Alexa Fluor 488 goat anti-rabbit IgG (A11008) was purchased from Life Technology (Eugene, OR, USA). PE-conjugated anti-mouse CD4 (clone RM4-5),PE/Cy7-conjugated anti-mouse CD8 (clone 53-6.7), FITC-conjugated anti-mouse CD25 (clone 3C7), Alexa Fluor 488-conjugated anti-mouseFoxP3 (clone 150D), APC-conjugated anti-mouse Gr-1 (clone RB6-8C5) and FITC-conjugated anti-mouse CD11b (clone M1/70) antibodies were purchased from BioLegend (San Diego, CA). PerCP-eFluor 710-conjugated anti-mouse CD3 (clone 17-A2)and APC eFluor 780-conjugatedanti-mouse CD127 (clone A7R34) antibodies were purchased from eBioscience (San Diego, CA).

### Chemotherapy administration

Cyclophosphamide (CTX) (10 mg/Kg), PTX (5 mg/Kg) and DTX (1 mg/Kg) diluted with phosphate-buffered saline (PBS) were administered via intraperitoneal injection (i.p.).The dose was extrapolated to human equivalent dose (HED) according to Reagan-Shaw et al. [30]. Chemotherapy was daily administered until the end of the experiment.

### In vitro cytotoxic effect

Mouse melanoma B16F10 cells were cultured for 12, 24 and 48 h at a concentration of 5 × 10^5^ cells per well in a 6-well plate in the presence of different concentrations of CTX, PTX and DTX. Drugs were added independently or as cocktail at indicated doses. Cells were collected, washed in PBS, pelleted and resuspened in 500 µl of PBS. Cell death was assessed by propidium iodide staining at flow cytometry using FACScan hardware using CellQuest software (BD Biosciences, Mountain View, CA).

### Calreticulin expression on the cell surface

Mouse melanoma B16F10 cells were cultured for 4 h at a concentration of 5 × 10^5^ cells per well in a 6-well plate in the presence of different concentrations of CTX, PTX and DTX. Drugs were added independently or as cocktail at indicated doses. Cells were collected, washed twice with PBS and fixed in 0.25 % of paraformaldehyde in PBS for 5 min. After washing again twice with cold PBS, cells were incubated for 30 min with anti-calreticulin (CRT) primary antibody diluted in cold blocking buffer (2 % FBS in PBS), followed by washing and incubation with the Alexa 488-conjugated monoclonal secondary antibody for 30 min. Each sample was analyzed by flow cytometry using FACScan hardware using CellQuest software (BD Biosciences, Mountain View, CA). Expression of CRT was evaluated on cell surface of propidium iodide negative cells. Untreated B16F10 melanoma cells were used as negative control.

### Subcutaneous tumor inoculation

Melanoma B16 cells were harvested in exponential growth phase by trypsinization and washed twice with ice-cold PBS, and then resuspended at a concentration of 1 × 10^6^ cells/ml. C57BL/6 mice were subcutaneously injected with 100 ul of B16 cells (1 × 10^5^ cells/mouse) on the right back flank. The tumor size was measured and documented every 3 days with a caliper, starting on day 7, and calculated using the formula (A × B^2^)/2 (A as the largest and B is the smallest diameter of tumor). Tumor growth was documented as mean tumor size with standard error disregarding single distant outliers. To record the survival of the tumor-bearing mice, either natural death or a tumor volume greater than 1600 mm^3^ leading to death was counted as death. Each experimental group included five animals.

### Characterization of regulatory T lymphocytes in whole blood, in spleens and in tumors

For Tregs detection whole blood samples of each mouse was directly staining with PE-conjugated anti-mouse CD4 and FITC-conjugated anti-mouse CD25 and incubated for 30 min at 4 °C. Following staining, whole blood was incubated with ACK lysing buffer for 7 min, washed with wash Medium (1 × PBS, 5 % FBS, 0.1 % NaN3), and after permeabilization, incubated with Alexa Fluor 488-conjugated anti-mouseFoxP3 for 30 min at 4 °C in the dark. The blood samples were collected and Tregs evaluated before mouse euthanasia.

After dissociation, splenocytes and tumor cells were incubated with ACK lysing buffer for 7 min, washed and resuspended in RPMI medium and incubated for 30 min at 4 °C in the dark with PE-conjugated anti-mouse CD4 and FITC-conjugated anti-mouse CD25 and APC eFluor 780-conjugatedanti-mouse CD127. After washing and permeabilization, cells were incubated with Alexa Fluor 488-conjugated anti-mouseFoxP3 for 30 min at 4 °C in the dark. To detect CD11b^+^/Gr-1^+^ myeloid-derived suppressor cells (MDSCs) from mouse splenocytes and single tumor cell preparations, cells were stained with APC-conjugated anti-mouse Gr-1 and FITC-conjugated anti-mouse CD11b antibodies.

The staining was characterized by flow cytometry using FACScan hardware using CellQuest software (BD Biosciences, Mountain View, CA).

### IFN-γ ELISpot assay

ELISPOT was performed according to BD Biosciences manufacturer instructions(BD ELISPOT Mouse IFN-γ ELISPOT Set cod. 551083). 2.5 × 10^5^ splenocytes were counted and plated in each well in duplicate. Cells were stimulated with 2 ug/ml of Trp2 peptide as well as with 5 ug/ml of phorbol myristate acetate (PMA, Sigma-Aldrich) and incubated for 24–26 h. As negative control PBS was used. The plates were read with an AID EliSpot Reader Systems (AID GmbH, Strassberg, Germany). The results were calculated as spot forming counts as a mean of a duplicate count from the specific antigen stimulation minus the negative control.

### Statistical analysis

Comparison between individual data points were performed with the unpaired two-sided Student’s *t* test. All P values were two-tailed and considered significant if less than 0.05.

## Results

### Cytotoxic effects in vitro of chemotherapy drugs

**Fig. 1:**
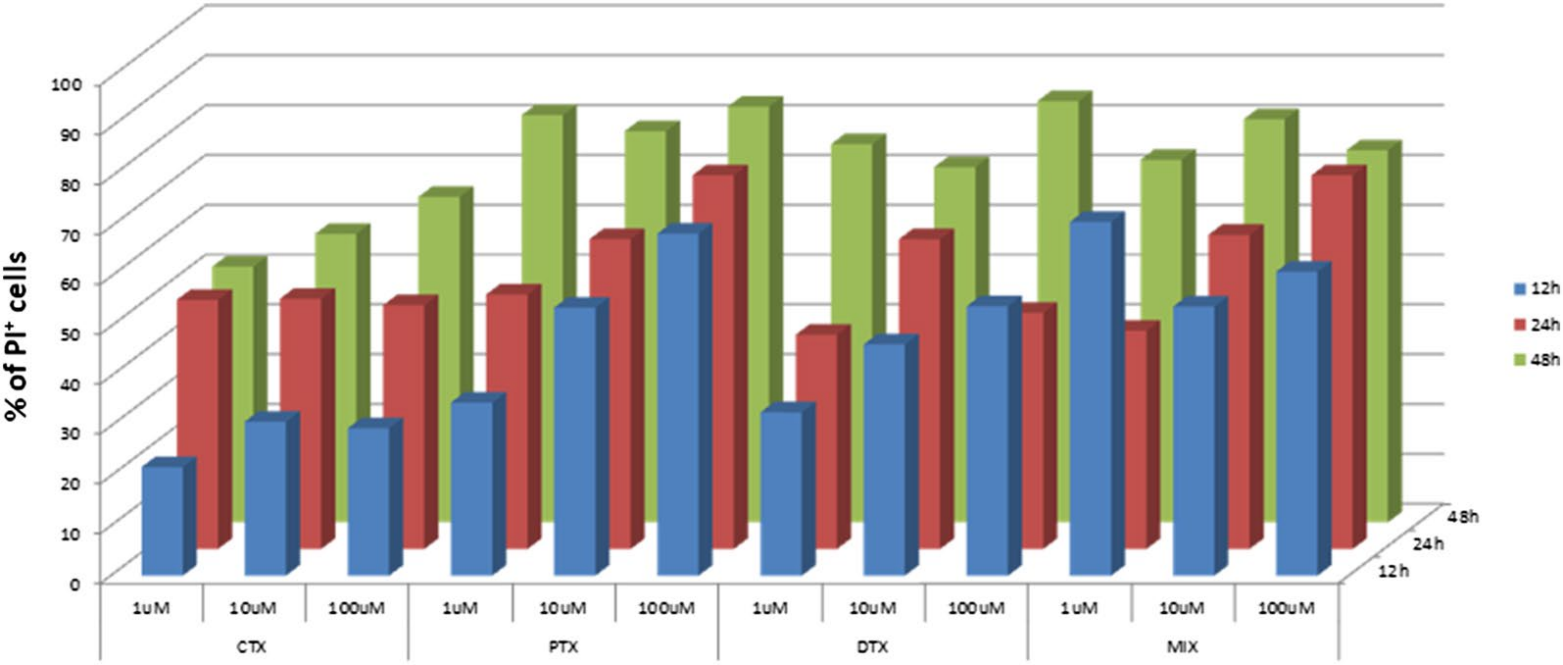
Mouse melanoma B16F10 cells were cultured for 12, 24 and 48 h at a concentration of 5 × 10^5^ cells per well in a 6-well plate in the presence of indicated concentrations of CTX, PTX, DTX and their mix. Cell death was assessed by propidium iodide staining at flow cytometry

Cytotoxic effects of CTX, DTX and PTX was assessed in vitro on B16F10 melanoma cells. Drugs were added to cell culture independently or as cocktail, in concentrations ranging from 1 to 100 µM. After incubation for 12, 24 and 48 h, cell death was assessed by flow cytometer cell counting, using the vital dye propidium iodide (PI). Results indicated that the three drugs induced a dose and time-dependent cell death, with different efficacy. Indeed, PTX and DTX showed a more pronounced cytotoxic effect than CTX. Moreover, the mix of the drugs induced a cumulative effect, mainly at lower doses and earlier time points (Fig. 1). Kinetic and morphological analyses indicated that morphologically discernible apoptosis was followed by secondary necrosis (data not shown).

### Calreticulin expression as marker of immunogenic cell death

**Fig. 2:**
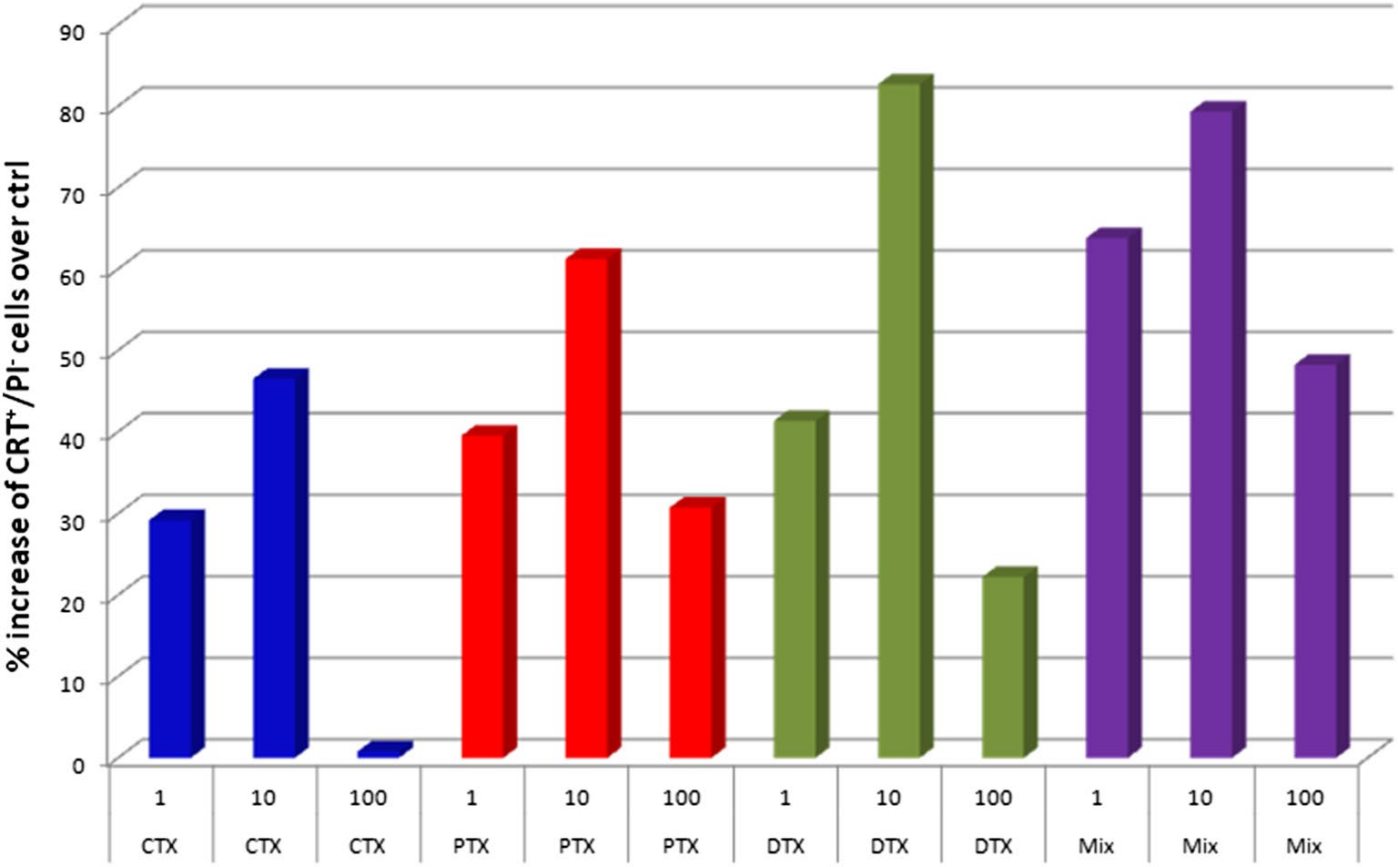
Mouse melanoma B16F10 cells were cultured for 4 h at a concentration of 5 × 10^5^ cells per well in a 6-well plate in the presence of indicated concentrations of CTX, PTX, DTX and their mix. Expression of CRT was evaluated on cell surface of propidium iodide negative cells. Values indicate the percentage of increase over the untreated B16F10 control cells

In order to assess whether CTX, PTX, DTX induced an immunogenic cell death, CRT expression was evaluated on the cell surface of B16F10 melanoma cells by flow cytometer after short-term stimulation (4 h). Results showed that the three drugs induced different levels of CRT expression on the surface of PI-negative cells. PTX and DTX induced a higher CRT expression than CTX. Moreover, the mix of the drugs induced a cumulative effect (Fig. 2). Interestingly, all drugs, alone or in the mix, induced the strongest effect at 10 µM concentration, with a significant reduction at 100 uM (Fig. 2).

### Effect of daily multi-drug metronomic chemotherapy on tumor growth and mice survival

**Fig. 3:**
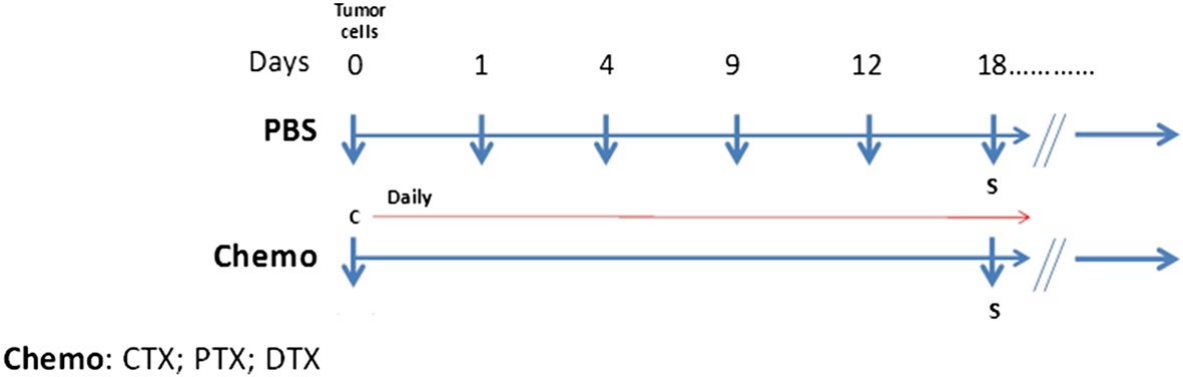
C57BL/6 mice were injected with 1 × 10^5^ cells in the right flank. The experimental group was treated daily in a metronomic fashion with a cocktail containing CTX (10 mg/Kg), PTX (5 mg/Kg) and DTX (1 mg/Kg)

In accordance to the in vitro results, a new multi-drug combination including CTX, DTX and PTX was designed to investigate its effect on tumor growth in vivo. C57BL/6 mice (five animals per group) were subcutaneously injected with B16F10 cells and daily treated in a low-dose metronomic fashion (Fig. 3).

The general status of animals in the experimental groups was followed up during the whole protocol. No toxicity was observed, all animals showing good general status without any significant weight loss during the whole treatment.

After injection of B16 tumor cells, animals were monitored every 3 days for tumor growth. Mice were sacrificed when tumor volume reached 1600 mm^3^ according to ethical rules.

**Fig. 4:**
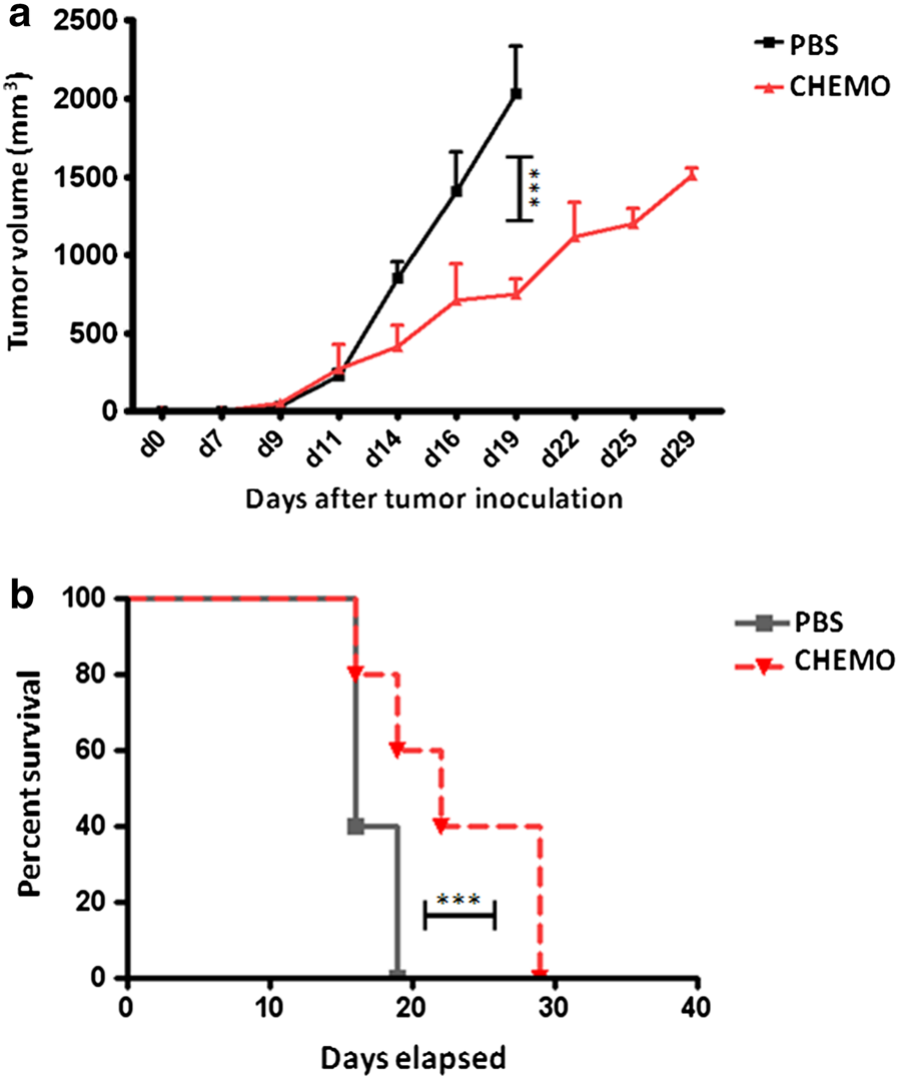
**a** Tumor growth was evaluated every three days with a caliper and tumor volume was calculated as indicated in Materials and Methods. Animals were sacrificed when tumor volume was greater than 1600 mm^3^. **b** Kaplan-Meyer curve showing the percentage of survival of animals in both experimental groups

The results showed that the metronomic chemotherapy significantly delays tumor growth as compared to the control group. At day 19, when all animals in both experimental groups were still alive and comparable, mice treated with metronomic chemotherapy showed a 70 % reduction in tumor dimension. Furthermore, even at the end of experiment, when all mice had to be sacrificed for ethical rules, the metronomic chemotherapy group showed tumor lesions with dimensions 30 % smaller than control group (Fig. 4a).

Consequently, the Kaplan-Meyer curve shows that, on average, the experimental group treated with daily metronomic chemotherapy had a significant prolonged survival (Fig. 4b).

### Effects of daily metronomic chemotherapy on T cell populations

Immunological analyses were performed on PBMCs, resected spleens and tumors from mice of both experimental groups.

**Fig. 5:**
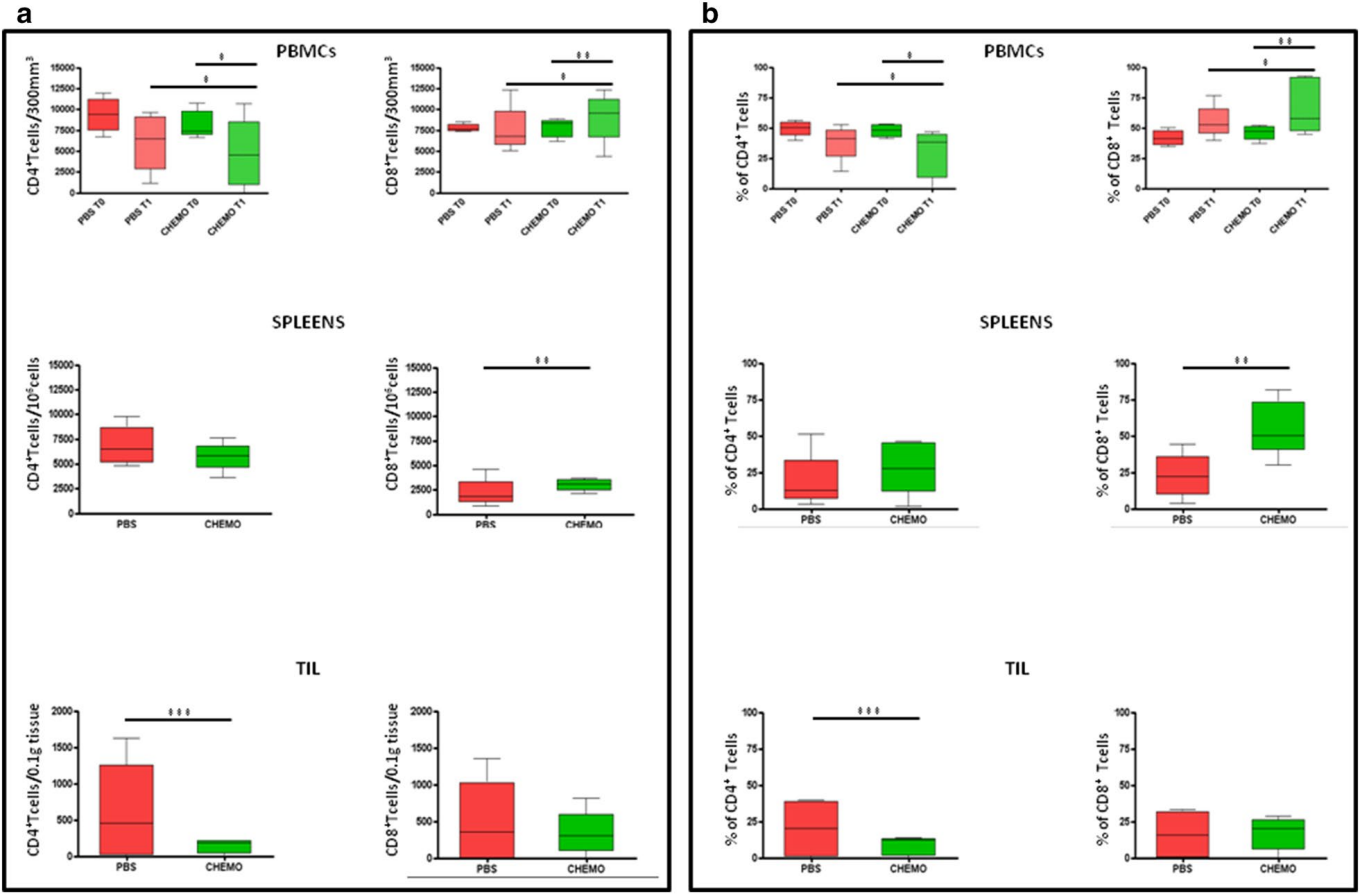
Evaluation of CD4^+^ and CD8^+^ T cells in PBMCs spleens and tumors in both experimental groups expressed as absolute numbers (**a**) and percentages (**b**). In PBMCs T0 represents the percentage at beginning of the protocol; T1 represents the percentage at the end of the protocol

Daily metronomic chemotherapy induced a reduction in the CD4^+^ T cells which was significant in PBMCS as well as in tumors, and a trend in the spleens. In parallel, CD8^+^ T cells significantly increased in PBMCs as well as in spleens, without significant change in the tumor (Fig. 5a, b). Nevertheless, the CD4^+^/CD8^+^ ratio did not drop below 1 which is the normal ratio in C57BL/6 mice at this age [31]. Such ratio is considered as marker of an immune competence status in HIV positive patients [32] (data not shown).

### Daily Metronomic chemotherapy reduces Treg population

In order to clarify the immunological mechanisms underlying the observed delay in tumor growth observed in the animal group treated with the daily metronomic chemotherapy, Tregs were evaluated in PBMCs, the spleen and the tumor microenvironment.

**Fig. 6:**
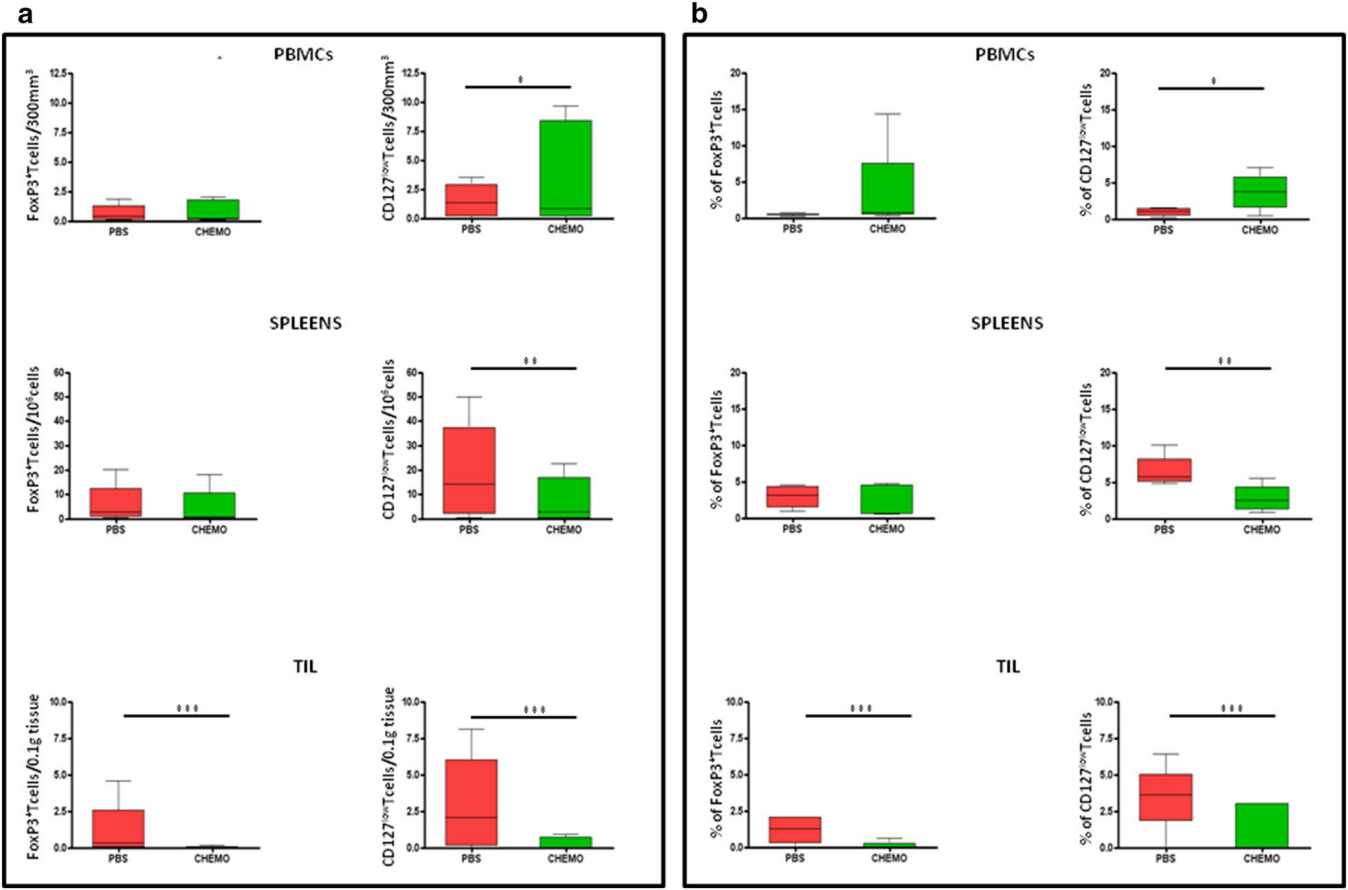
Evaluation of CD4^+^ CD25^+^FoxP3^+^ and CD4^+^ CD25^+^CD127^low^ T regulatory cells in PBMCs, spleens and in the tumor of animals in both experimental groups expressed as absolute numbers (**a**) and percentages (**b**)

The results showed a significant reduction in CD4^+^CD25^+^FoxP3^+^ as well as CD4^+^CD25^+^CD127^low^ Treg populations in both spleens and tumors of mice treated with daily metronomic chemotherapy as compared to untreated groups. On the contrary, Tregs were not reduced but even increased in PBMCs in treated animals as compared to control group (Fig. 6a and b).

**Fig. 7:**
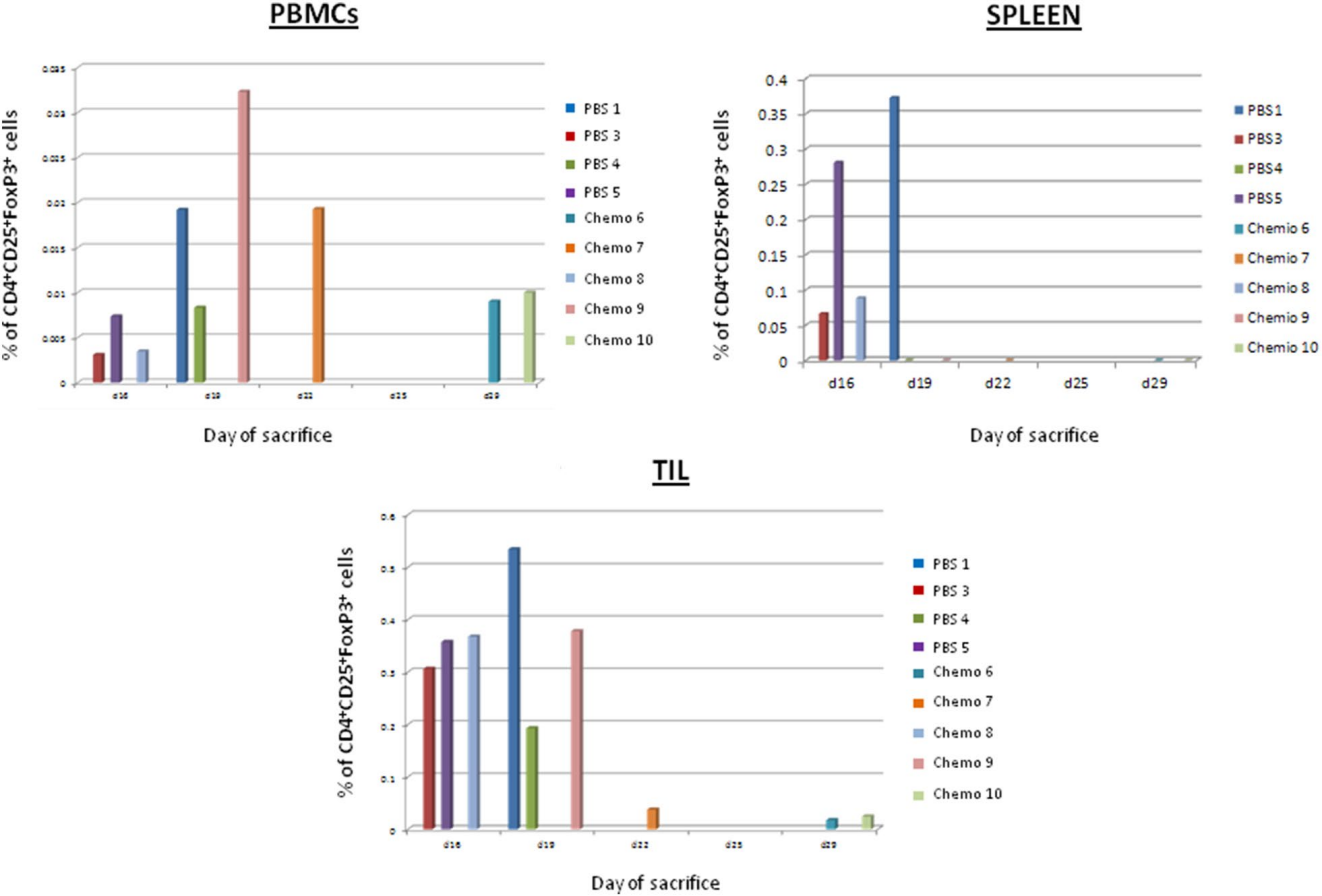
Correlation analysis between day of sacrifice of each animal and percentage of CD4^+^ CD25^+^FoxP3^+^ T regulatory cells in PBMCs, spleens and in the tumor

**Fig. 8:**
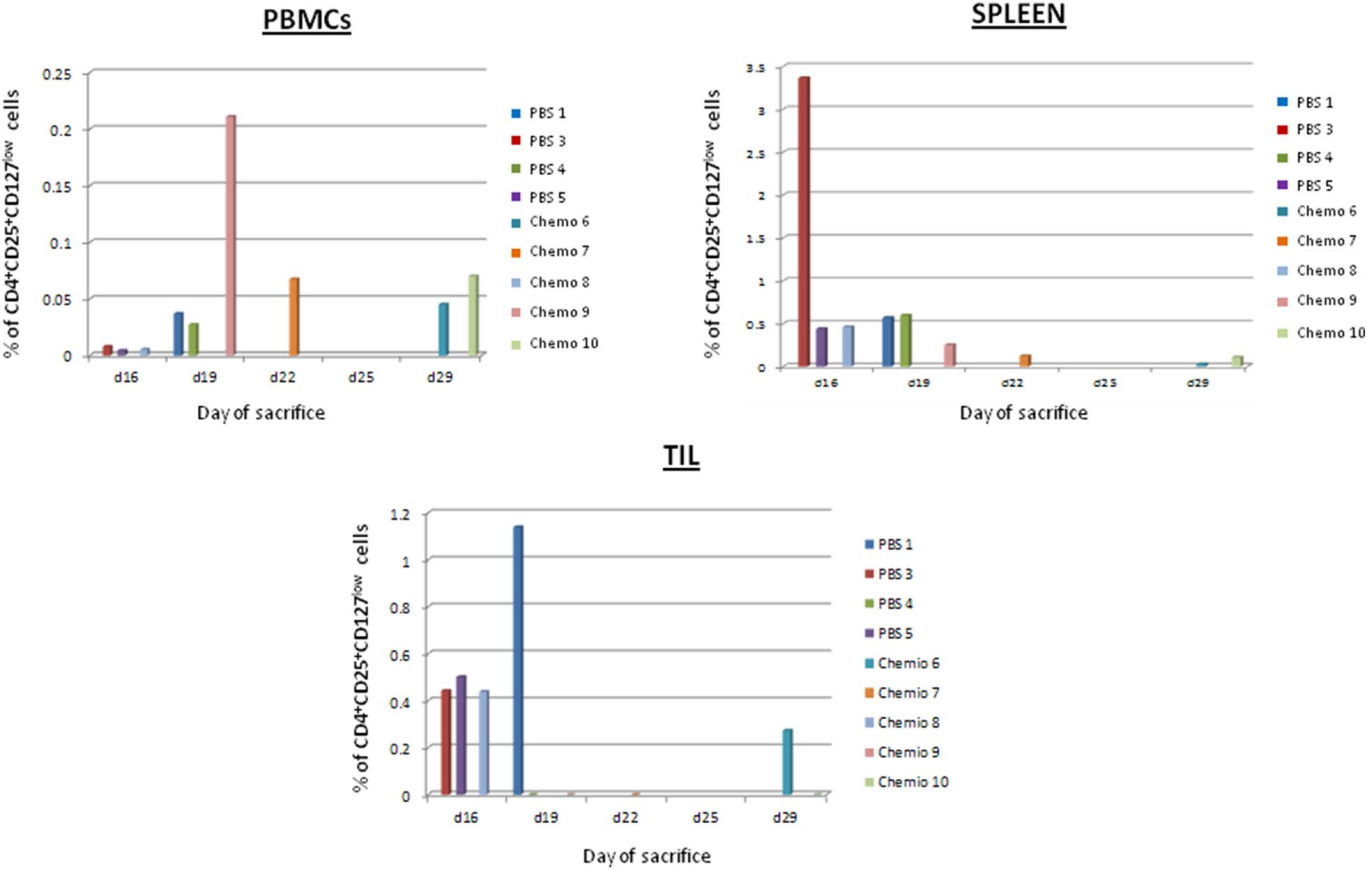
Correlation analysis between day of sacrifice of each animal and percentage of CD4^+^ CD25^+^CD127^low^ T regulatory cells in PBMCs, spleens and in the tumor

In order to verify whether the percentage of intra-splenic and/or intra-tumor Tregs population correlated with tumor growth and survival, a correlation analysis was performed. Results showed a strong inverse correlation between percentage of intra-splenic and/or intra-tumor Tregs population with tumor growth and survival. Indeed, the lowest percentage of Tregs was observed in mice showing the slowest tumor growth and the longest survival (p < 0.035) (Figs. 7, 8).

### Evaluation of IFN-γ producing T cells

**Fig. 9:**
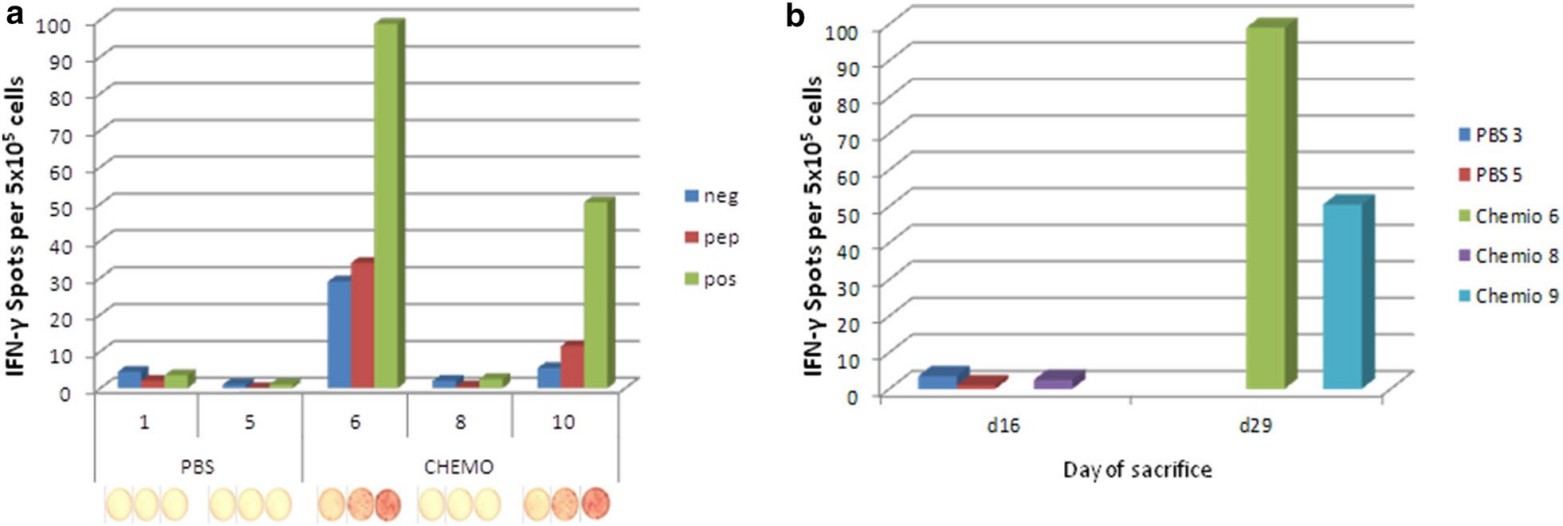
**a** Results of IFN-γ ELISPOT obtained with splenocytes from individual animals. **b** Correlation analysis between day of sacrifice of each animal and number of spots. Neg = control peptide; pep = Trp2 peptide; pos = PMA

Splenocytes were further assessed for IFN-γ production in an Elispot assay. Upon re-stimulation with tumor-associated Trp2 peptide, only splenocytes from mice treated with metronomic chemotherapy showed a significant IFN-γ production (Fig. 9). Interestingly, this group showed also a relatively high IFN-γ production at baseline as well as upon PMA stimulation, suggesting a general T cell activation induced by the chemotherapy.

A direct correlation between levels of IFN-γ production and survival was observed (p < 0.023), to confirm that such responsive T cells play a key role in the observed delay of tumor growth and increased animal survival.

## Conclusion

In the current study, the immunomodulatory effect of a daily administered novel multi-drug metronomic chemotherapy was evaluated in a tumor bearing mouse model.

In particular, the metronomic chemotherapy consisted of a multi-drug cocktail including taxanes (docetaxel and paclitaxel) and alkylating (cyclophosphamide) agents, to hit different targets of the immune suppressive environment.

The results of ex vivo treatment of B16F10 cells with drugs showed that they have different cell toxicity and properties in inducing an immunological cell death. This represents the required effect in order to provide immunogenic signals and induce an effective anti-tumor immunity. In particular, the taxanes showed the most potent activity. Moreover, the drug mix significantly potentiated the individual activities, supporting the use in the in vivo animal experiments.

The combined metronomic chemotherapy was administered in a daily schedule in C57BL/6 animal model injected with the highly aggressive B16F10 melanoma cell line.

The treatment induced a significant reduction in tumor growth and prolonged survival as compared to the control group (Fig. 2a, b). Such effect was directly correlated with reduction of CD4^+^ Tcells and parallel increase of CD8^+^ Tcells (Fig. 3). CD4^+^CD25^+^FoxP3^+^as well as CD4^+^CD25^+^CD127^low^ Treg populations were found significantly reduced in both spleens and tumors of sacrificed mice treated with daily metronomic chemotherapy as compared to untreated groups (Fig. 4). The percentage of intra-splenic and/or intra-tumor Treg populations, and not in PBMCs, showed a strong inverse correlation with tumor growth and survival. Indeed, the lowest percentage of Tregs was observed in mice showing the slowest tumor growth and the longest survival (Figs. 5, 6). Such findings indicating that the effects of daily metronomic chemotherapy was directly correlated with a sustained reduction in the Treg population in spleens and in the tumor, as previously reported [3, 21, 26, 33–40].

Unlike the observed reduction in spleens and tumors, Tregs level in PBMCs of treated animals were similar or even higher as compared to untreated groups. Such a discrepancy has been reported also by others [41]. This could be explained by the observation that the lymphocyte recovery after the lymphopenia induced by chemotherapy results in increased Treg proliferation [42]. Indeed, Tregs show a higher regeneration rate compared to Teffs (8 days vs, 24–199 days) [43]. This would allow Tregs to return to their normal cell cycling and growth in the germinal centers and their rapid re-enter in circulation. Overall, such a finding confirms that evaluation of circulating Trges is not representative of the intratumoral microenvironment.

IFN-γ producing T cells were found to be significantly higher in splenocytes of treated animals as compared to control group (Fig. 7). Such result was observed not only upon ex vivo re-stimulation with tumor-associated Trp2 peptide but also at baseline as well as upon PMA stimulation. Also for this parameter, a direct correlation between levels of IFN-γ production and survival was observed, to confirm that such responsive T cells play a key role in the observed in vivo effects.

The described results suggest that the chemotherapy exerts a two-pronged activity, both inducing an “immunological death” of cancer cells and a general T cell activation, resulting in delayed tumor growth and increased survival.

Overall, these results provide a rationale for the use of the novel multi-drug chemotherapeutic combination to overcome the immunesuppressive tumor microenvironment. Such effect would eventually result in dramatic improvement in the intrinsic immune containment of tumor growth as well as the immune response elicited by immunotherapy strategies. To this aim, a combinatorial strategy including the described multi-drug metronomic chemotherapy and cancer vaccines will provide further experimental evidences to confirm such observation.
